# Supplementary material for: Green light triggered [2+2] cycloaddition of halochromic styrylquinoxaline—controlling photoreactivity by pH
Source: Nat Commun. 2020 Aug 21;11:4193. doi: 10.1038/s41467-020-18057-9 (PMC7443129; doi:10.1038/s41467-020-18057-9)
Supplement: Supplementary file 1 — Supplementary Information [file 41467_2020_18057_MOESM1_ESM.pdf]

Supplementary Information

Green Light Triggered [2+2] Cycloaddition of a Halochromic Styrylquinoxaline – Controlling Photoreactivity by pH

Kalayci et al.

Table of Contents

1. Supplementary Figures 3

2. Supplementary Methods 9

2.1. SEC-ESI-MS 9

2.2. DMAC-SEC 9

2.3. ^{1}H NMR Measurements 9

2.4. Chromatography 9

2.5. Mass Spectrometry 9

2.6. UV-VIS Spectroscopy 10

2.7. Rheology 10

2.8. Photochemical Procedures 10

2.8.1 Experiments using LED lamps 10

2.8.2 Experiments using laser irradiation 10

2.8.3 Dimerization of PEG-SQ 11

2.8.4 Control over the constant number of photons 11

2.8.5 Transmittance of the glass vials 12

2.8.6 Calculation of conversions for action plot 13

2.9. Hydrogel swelling 13

2.10. Cell culture study 13

2.11. Synthetic procedures 14

2.11.1 Materials 14

2.11.2 Synthesis of ethyl 4-(4-formyl-2-methoxyphenoxy) butanoate (S1) 14

2.11.3 Synthesis of 2-methylquinoxaline (S2) 14

2.11.4 Synthesis of ethyl (E)-4-(2-methoxy-4-(2-(quinoxalin-2-yl)vinyl)phenoxy)butanoate (S3) 15

2.11.5 Synthesis of (E)-4-(2-methoxy-4-(2-(quinoxalin-2-yl)vinyl)phenoxy)butanoic acid (S4) 16

2.11.6 Synthesis of 2,5-dioxopyrrolidin-1-yl (E)-4-(2-methoxy-4-(2-(quinoxalin-2-yl)vinyl)phenoxy)butanoate (S5) 17

2.11.7 Synthesis of 8-arm PEG-NH_2 19

2.11.8 Synthesis of linear MeO-PEG-styrylquinoxaline (PEG-SQ) and 8arm-PEG- styrylquinoxaline (8arm $(PEG\text{-}SQ)_8$) 20

3. Supplementary References 22

1. Supplementary Figures

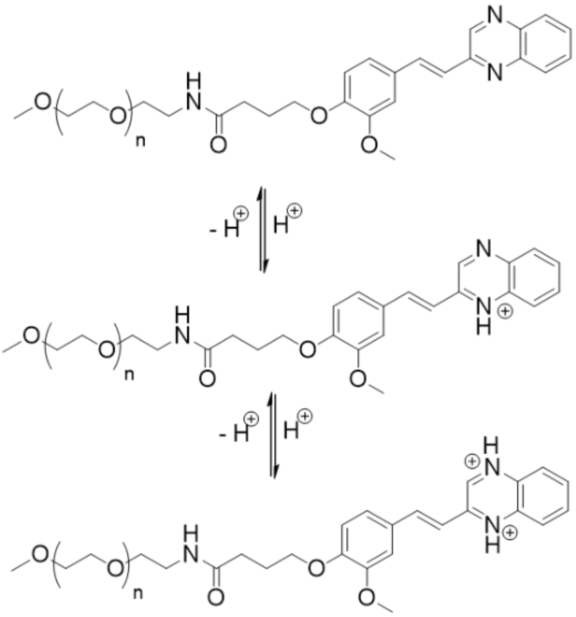

Supplementary Figure 1. Proposed mechanism of protonation of the styrylquinoxaline in acidic condition, which gives rise to different UV/vis absorption spectra and consequently photo-reactivity.

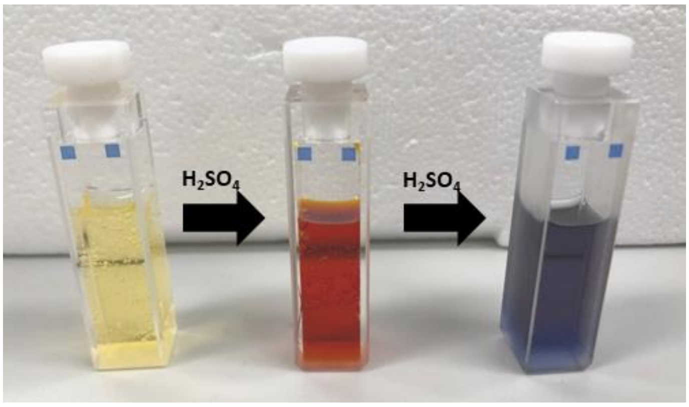

Supplementary Figure 2. Photographic image showing the colour change of a **PEG-SQ** solution (10 mg mL^{-1}) after addition of 0.1 mL concentrated H_2SO_4 in each step.

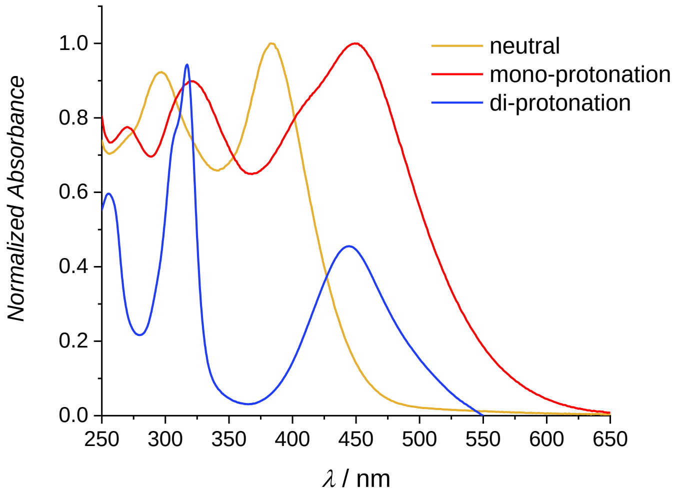

Supplementary Figure 3. UV/vis spectra of **PEG-SQ** (10 mg mL^{-1}) solutions after addition of 0.1 mL concentrated H_2SO_4 for each protonation step.

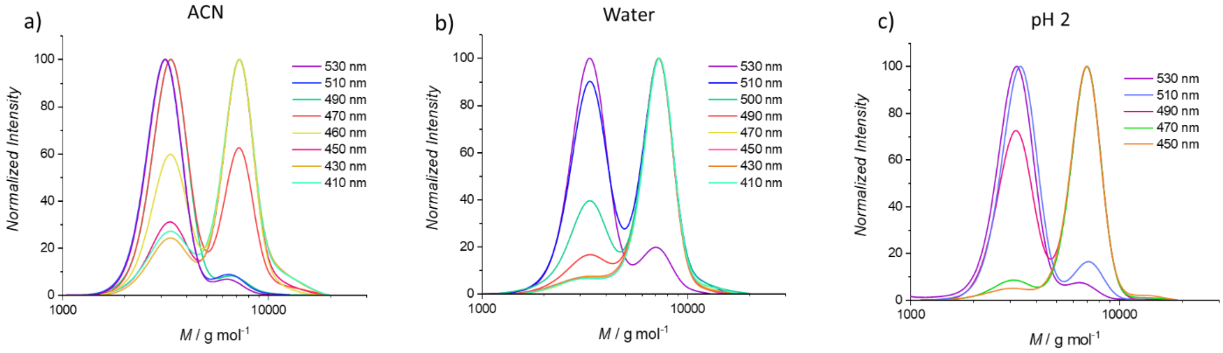

Supplementary Figure 4. SEC data showing conversion of **PEG-SQ** to **(PEG-SQ)**$_2$ after irradiation at different wavelengths with the same number of photons and concentration ($1.23 \cdot 10^{22}$ photons, concentration = 10 mg mL^{-1}) **a)** in acetonitrile, **b)** in water and **c)** at pH 2.

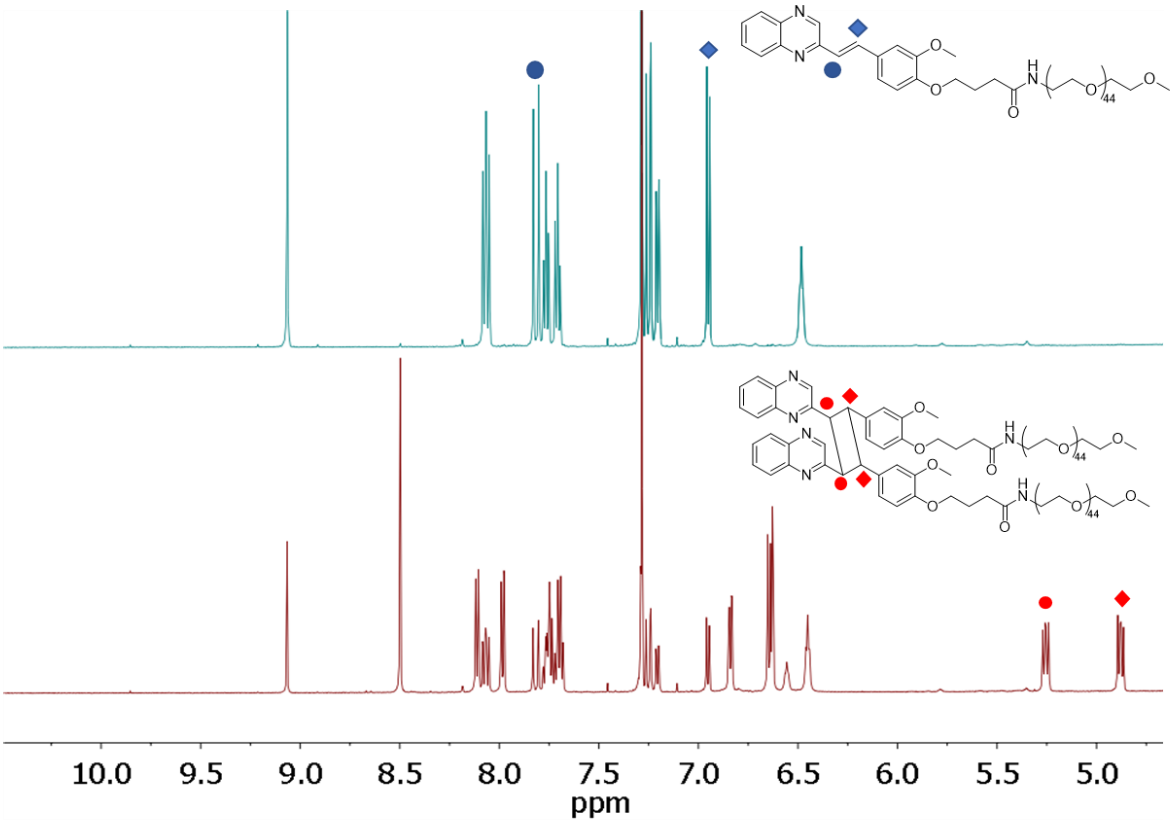

Supplementary Figure 5. ^{1}H NMR spectra ($CDCl_3$, 400 MHz) of **PEG-SQ** before (**top**) and after (**bottom**) irradiation with green light (510 nm, 20 mW cm^{-2}) for 1 h, showing ca. 75% conversion to dimer. The conversion was calculated from the integration of the alkene chemical shifts and cyclobutane adduct chemical shifts.

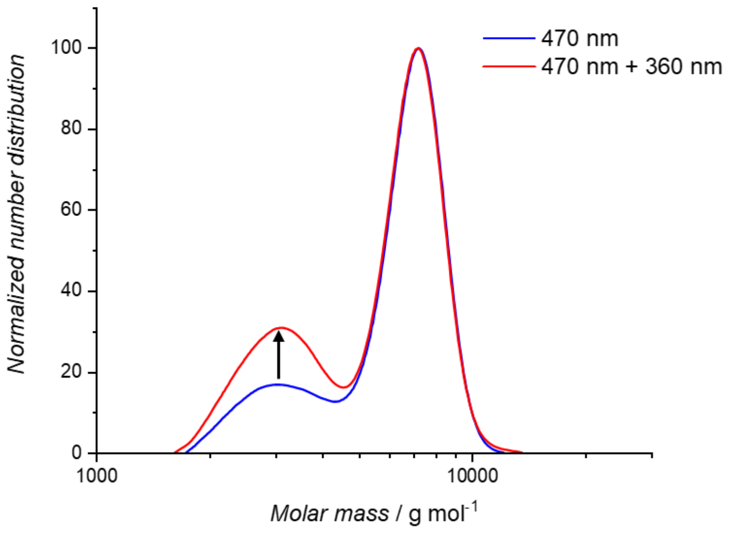

Supplementary Figure 6. SEC data of **PEG-SQ** samples (10 mg mL^{-1}) after irradiation at 470 nm with 1.23·10^{22} photons and subsequent irradiation at 360 nm in water.

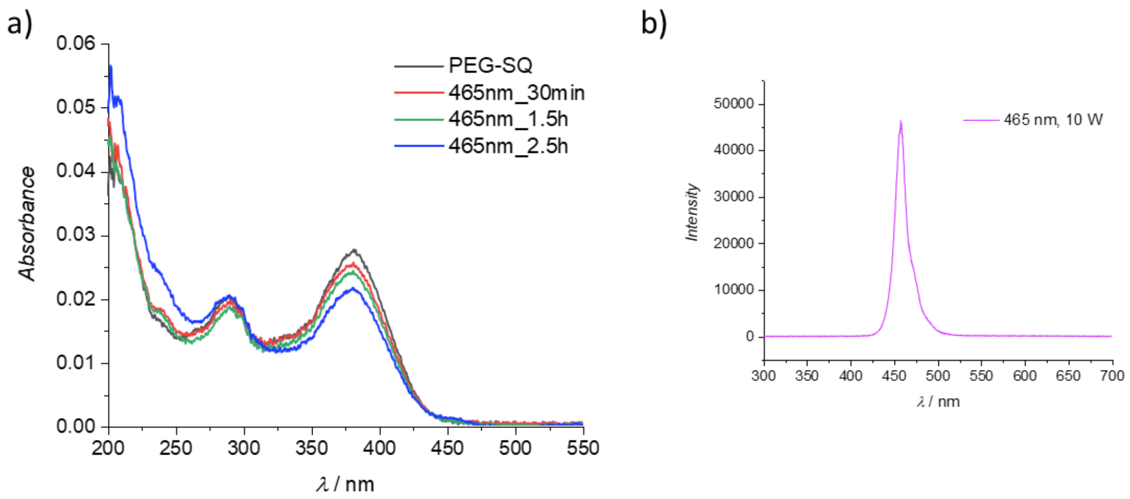

Supplementary Figure 7. a) UV/vis spectra of PEG-SQ solution in ACN ($2.5 \cdot 10^{-3}$ mg mL^{-1}) after irradiation with 465 nm LED (10 W). **b)** Emission spectrum of the LED (465 nm, 10 W).

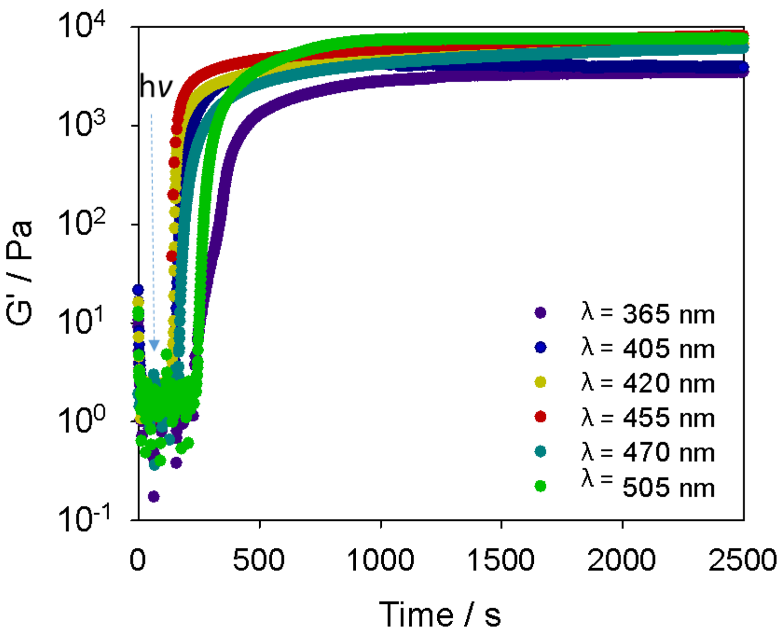

Supplementary Figure 8. Full gelation profiles, followed by rheology, of polymer 8arm **$(PEG\text{-}SQ)_8$** (c = 5 mM) under irradiation at different wavelength.

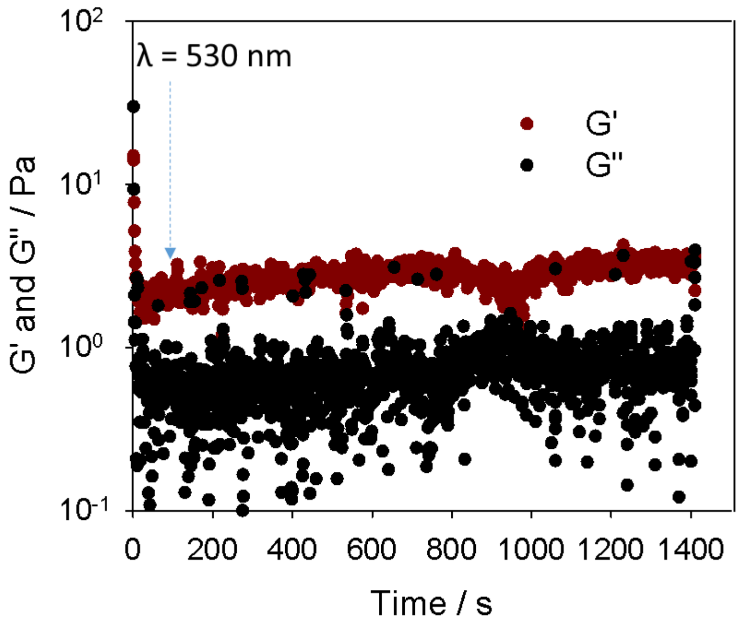

Supplementary Figure 9. Gelation profiles, followed by rheology, of 8arm **(PEG-SQ)$_8$** (c = 5 mM) under irradiation at λ = 530 nm.

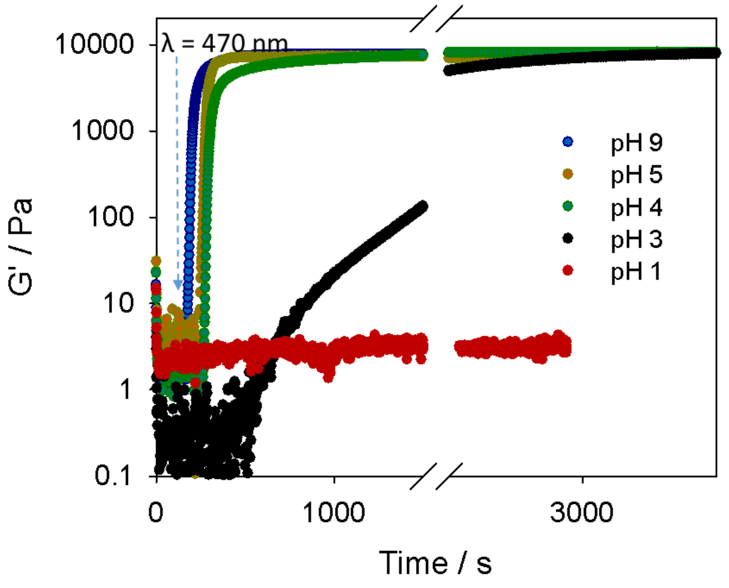

Supplementary Figure 10. Full gelation profiles, followed by rheology, of polymer 8arm **(PEG-SQ)$_8$** (c = 5 mM) under irradiation at λ = 470 nm and different pH.

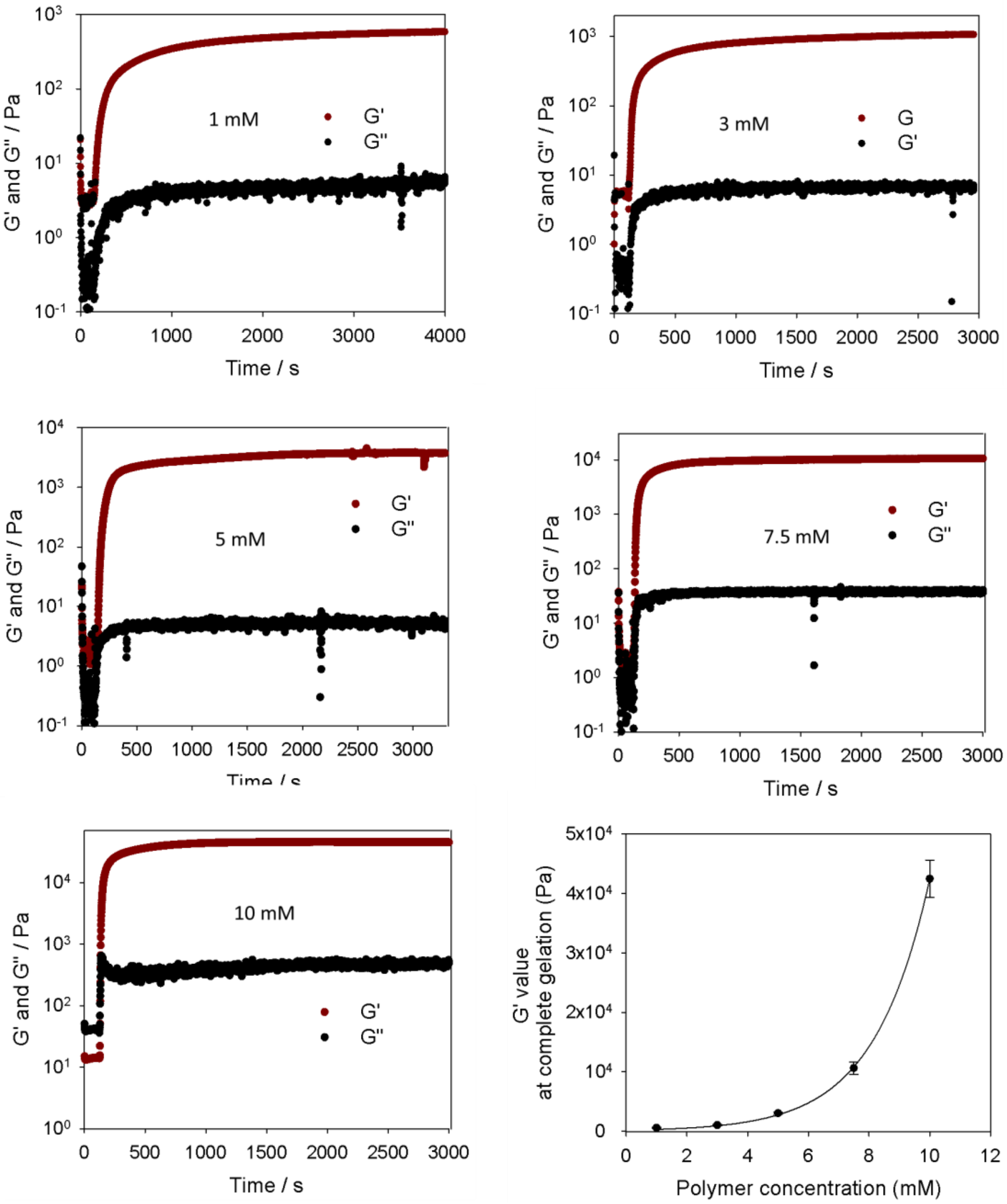

Supplementary Figure 11. Gelation profile of 8-arm **(PEG-SQ)$_8$** at different polymer concentrations and the G’ values at complete gelation (n = 3).

2. Supplementary Methods

2.1. SEC-ESI-MS

Spectra were recorded on a Q Exactive Plus (Orbitrap) mass spectrometer (Thermo Fisher Scientific, San Jose, CA, USA) equipped with a HESI II probe. The instrument was calibrated in the m/z range 74-1822 using premixed calibration solutions (Thermo Scientific) and for the high mass mode in the m/z range of 600-8000 using ammonium hexafluorophosphate solution. A constant spray voltage of 3.5 kV, a dimensionless sheath gas and a dimensionless auxiliary gas flow rate of 10 and 0 were applied, respectively. The capillary temperature was set to 320 °C, the S-lens RF level was set to 150 and the aux gas heater temperature was set to 125 °C. The Q Exactive was coupled to an UltiMate 3000 UHPLC System (Dionex, Sunnyvale, CA, USA) consisting of a pump (LPG 3400SD), autosampler (WPS 3000TSL), and a temperature-controlled column department (TCC 3000). Separation was performed on two mixed bed size exclusion chromatography columns (Agilent, Mesopore 250 × 4.6 mm, particle diameter 3 µm) with a precolumn (Mesopore 50 × 7.5 mm) operating at 30 °C. THF at a flow rate of 0.30 $mL \cdot min^{-1}$ was used as eluent. The mass spectrometer was coupled to the column in parallel to an UV-detector (VWD 3400, Dionex), and a RI-detector (RefractoMax520, ERC, Japan) in a setup described earlier.[1] 0.27 $mL \cdot min^{-1}$ of the eluent were directed through the UV- and RI-detector and 30 $\mu L \cdot min^{-1}$ were infused into the electrospray source after post-column addition of a 50 µM solution of sodium iodide in methanol at 20 $\mu L \cdot min^{-1}$ by a micro-flow HPLC syringe pump (Teledyne ISCO, Model 100DM). A 200 µL aliquot of a polymer solution with a concentration of 2 $mg \cdot mL^{-1}$ was injected into the SEC system.

2.2. DMAC-SEC

The SEC measurements were conducted on a PSS SECurity system consisting of a PSS SECurity Degasser, PSS SECurity TCC6000 Column Oven (60 °C), PSS GRAM Column Set (8x150 mm 10 µm Precolumn, 8x300 mm 10 µm Analytical Columns, 1000 Å, 1000 Å and 30 Å) and an Agilent 1260 Infinity Isocratic Pump, Agilent 1260 Infinity Standard Autosampler, Agilent 1260 Infinity Diode Array and Multiple Wavelength Detector (A: 25415 nm, B: 360 nm), Agilent 1260 Infinity Refractive Index Detector (35 °C). HPLC grade DMAc, 0.01 M LiBr, is used as eluent at a flow rate of 1 $mL \cdot min^{-1}$. Narrow disperse linear poly (methyl methacrylate) (Mn: 202 $g \cdot mol^{-1}$ to 2.2×106 $g \cdot mol^{-1}$) standards (PSS ReadyCal) were used as calibrants. All samples were passed over 0.22 µm PTFE membrane filters. Molecular weight and dispersity analysis were performed in PSS WinGPC UniChrom software (version 8.2).

2.3. ^{1}H NMR Measurements

NMR spectra were recorded on a Bruker Avance III 400 MHz or 600 MHz with a 5 mm broadband auto-tunable probe with Z-gradients at 293 K. Chemical shifts are reported as δ in parts per million (ppm) and referenced to the chemical shift of the residual solvent resonances ($CDCl_3$ δ = 7.26 ppm), couplings are shown as s: singlet, d: doublet, t: triplet, m: multiplet. Polymer samples were prepared at a concentration of 10 mg mL^{-1}. In most spectra traces of water appears as a broad singlet at around 1.5-2.5 ppm. NMR spectra were processed using MestReNova software.

2.4. Chromatography

Thin-layer chromatography (TLC) was performed on silica gel 60 F254 alumina sheets (Merck) and visualized by UV light or potassium permanganate solution. Column chromatography was run on silica gel 60 (0.04-0.06 mm, 230-400 mesh ASTM, Merck).

2.5. Mass Spectrometry

Test compounds were infused directly into the MS via a kdScientific infusion pump at a static flow rate of 650 µL/h. MS setup was as followed: Agilent 6220 TOF MS system (Santa Clara, CA, USA) with a multimode dual nebuliser ESI/APCI source. The MS was operated in positive or negative mode using the following conditions: nebulizer pressure 35 psi, gas flowrate 8 L/min, gas temperature 300°C,

capillary voltage 2500/-2500 V, fragmentor 150 and skimmer 65 V. The instrument was operated in the extended dynamic range mode with data collected in m/z range 100–3200.

2.6. UV-VIS Spectroscopy

UV/vis spectra were recorded on a *Shimadzu* UV-2700 spectrophotometer equipped with a CPS-100 electronic temperature control cell positioner. Samples were prepared in THF and measured in *Hellma Analytics* quartz high precision cells with a path length of 10 mm at ambient temperature.

2.7. Rheology

Rheological experiments were studied using an Anton Paar Physica rheometer with a plate-plate configuration. The lower plate is made of quartz and the upper plate is made of stainless steel with a diameter of 15 mm. A liquid light guild, which was connected to the WheeLED light source, was equipped below the quartz plate. In a typical experiment, 50 µL of a solution of **P2** was placed on the lower plate and the upper plate was brought to a measurement gap of 0.2 mm. A layer of paraffin oil was applied on the edge of the stainless-steel plate to prevent dehydration of hydrogel and the test was started by applying a 1% strain with the frequency of 0.1 Hz on the sample.

2.8. Photochemical Procedures

2.8.1 Experiments using LED lamps

Light irradiation for polymer crosslinking was performed using a Mightex's WheeLED wavelength-switchable LED for λ = 405-505 nm (built-in filters). The intensity of light irradiance on the gel sample was tuned to the desired intensity (20 mW cm^{-2}) using a RM-12 radiometer (Opsytec) with a sensor VISBG 400-570 nm.

2.8.2 Experiments using laser irradiation

The incident light used for laser experiments was a Coherent Opolette 355 tunable OPO operated at 410-500 nm with a full width half maximum of 7 ns and a repetition rate of 20 Hz. The emitted pulse, which has a flat-top spatial profile, was expanded to 6mm diameter using focusing lenses and directed upwards using a prism. The beam was then centered on a glass laser vial which is positioned in a 6mm diameter slot in a temperature-controlled sample holder. The energy transmitted through the sample holder was measured using a Coherent Energy Max PC power meter.

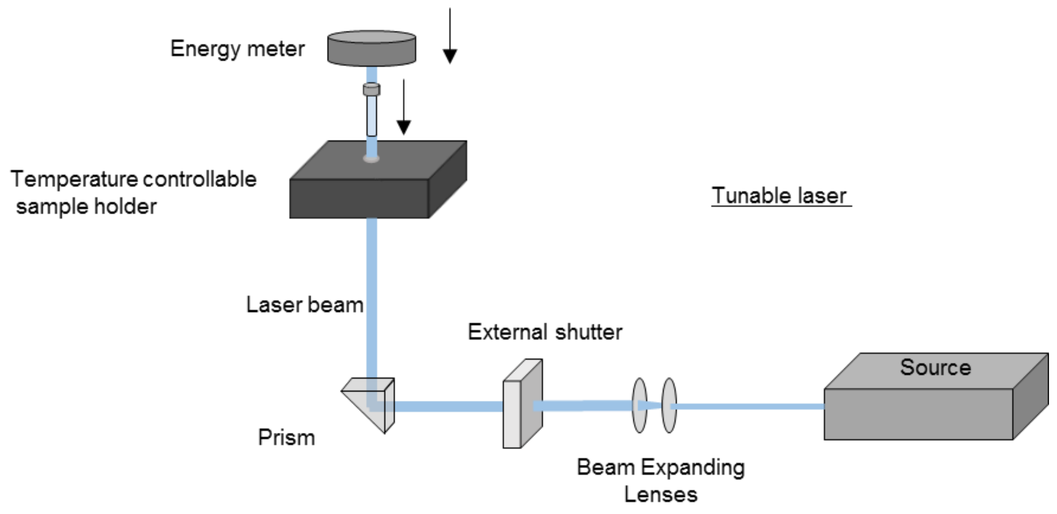

Supplementary Figure 12. Experimental setup for tunable laser experiments.

2.8.3 Dimerization of PEG-SQ

PEG-SQ (1 mg) was dissolved in 0.1 mL of Acetonitrile or water in laser vials, the vials were crimped airtight and degassed for 5 min applying Argon. Each solution was irradiated at λ = 410-550 nm with the same number of photons.

2.8.4 Control over the constant number of photons [2]

The number of photons n_p ($[n_p]$ = mol) that a monochromatic laser pulse contains can be calculated by application of the Planck-Einstein relation from the energy of the pulse E_{pulse}, the incident wavelength λ, Planck's constant h and the speed of light c:

$$n_p = \frac{E_{pulse}\ \lambda}{h\ c\ N_A} \quad (1)$$

If the absorption of the glass vial and the extent of reflection and scattering at the vial at the respectively relevant wavelength is known, a target energy value can be calculated that must be reached during the above described measurement to guarantee that the desired number of photons penetrates the sample solution during the subsequent irradiation. The wavelength dependent transmittance of the glass vials was determined experimentally using the above setup. Three glass vials were randomly selected as calibration vials. For varying wavelengths and in each case at a constant power output of the laser the energy was measured both with and without the calibration vials fitted into the sample holder. The top parts of these vials were cut off to minimize errors in the procedure, since only the bottom and sides of the glass vials would contribute significantly to the reduction of the photon flux that enters the solution.

The measured energy per pulse without a calibration vial in the sample holder is denoted as E_0 and the measured energy per pulse with a calibration vial in the sample holder as E_n. The transmittance was calculated as the ratio of E_n to E_0. The average transmittance over the measurements of the three vials (T_λ) was plotted together with the respective error (compare7):

$$T_\lambda = \frac{E_n}{E_0} \quad (2)$$

The target energy per pulse E_0 can be calculated directly from the wavelength λ, the number of pulses k, the transmittance of the glass vial at the respective wavelength T_λ and the desired total photon count n_p:

$$E_0 = \frac{n_p\ N_A\ h\ c}{k\ T_\lambda\ \lambda} \quad (3)$$

By controlling the target E_0 at the respective wavelength, the number of photons that penetrate each sample solution of one set of experiments as described in the following subsections was guaranteed to be identical despite irradiation at different wavelengths.

An example calculation (λ = 300 nm) is shown below:

$$E^0 = \frac{2.5\cdot10^{-7}\ \text{mol}\cdot6.022141\cdot10^{23}\ \text{mol}^{-1}\cdot6.62607\cdot10^{-34}\ \text{kg}\frac{\text{m}^2}{\text{s}}\cdot2.99792\cdot10^{8}\frac{\text{m}}{\text{s}}}{1000\cdot0.377\cdot3\cdot10^{-7}\ \text{m}} = 2.64\cdot10^{-4}\ \text{J} \quad (4)$$

2.8.5 Transmittance of the glass vials

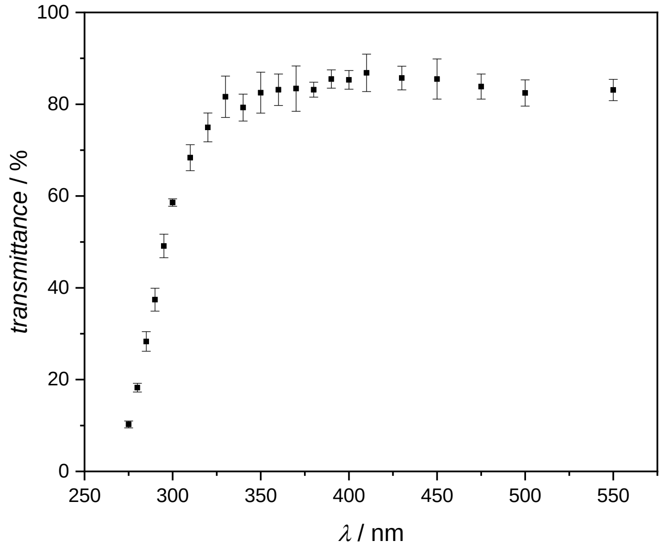

Supplementary Figure 13. Transmittance of glass vials in dependence of irradiation wavelength, refer to Supplementary Table 1.

Supplementary Table 1. Transmittance of the bottom of the glass vials used in this study. The transmittance values shown and used here were obtained analogously to a method reported previously.[3] The glass vials were cut at a height of 3 mm. Thus, the number of photons delivered into the sample solution can be determined more precisely, than in initial attempts to estimate the number of photons.[4] The values are in agreement to the previously found transmittance at 285 nm, which was determined with the same glass vials as used here.[5]

λ / nm	T_λ / %	Standard deviation
275	10.23419	0.74982
280	18.25024	0.94703
285	28.31044	2.14137
290	37.43074	2.49132
295	49.11881	2.55629
300	58.57746	0.79725
310	68.34923	2.82282
320	74.95204	3.14492
330	81.62937	4.49571
340	79.27099	2.93382
350	82.52063	4.45387
360	83.15709	3.42489
370	83.40189	4.93431
380	83.17113	1.63659
390	85.48502	1.99615
400	85.31216	2.03069
410	86.83816	4.06669
430	85.71046	2.57226
450	85.49291	4.38069
475	83.84506	2.73637
500	82.44994	2.85985
550	83.10058	2.33119

2.8.6 Calculation of conversions for action plot

The conversion of the dimerization reactions was calculated from the SEC data (Supplementary Figure 4). Initially, the integral of the peaks was calculated by applying Gaussian formulation for the peak fitting. Then the below equation was used to obtain the conversion as percentage where A_1 is the area under first peak (PEG-SQ) and A_2 is the area under second peak ((PEG-SQ)$_2$).

$$\%\,Conversion = \frac{A_2}{(A_1+A_2)} \cdot 100 \qquad (5)$$

2.9. Hydrogel swelling

To study the effect of pH on hydrogel swelling, a polymer solution (c = 7.5 mM, 200 µL) in a 5 mL sealed vial was irradiated with light at 455 nm for 3 h. The resultant solid gel was extracted from the vial and placed in excess PBS solution pH 7.4. The weight of the hydrogel was monitored until no further change in the weight was observed. The pH of the solution was subsequently adjusted using HCl 1 M and NaOH 1 M solution. At each set pH, the weight of the hydrogel (w_t) was recorded when no further change in the weight was observed. The swelling ratio was calculated by:

$$Q = \frac{w_t}{w_0} \qquad (6)$$

Where w_0 is the weight of as-prepared hydrogel. The experiment was done in triplicate.

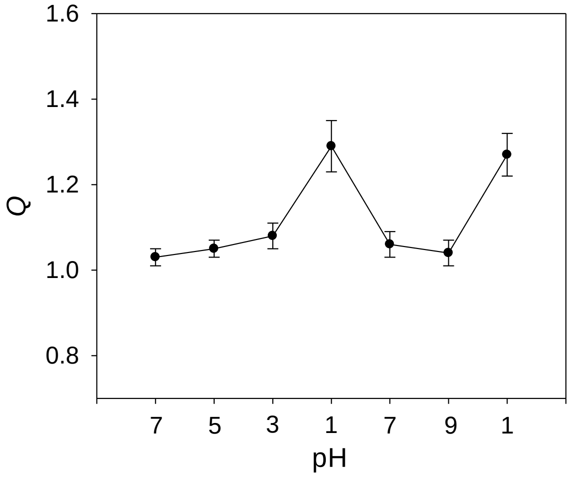

Supplementary Figure 14. pH-dependent swelling ratio (*n* = 3) of hydrogel prepared from 10 wt% solution and under irradiation at 455 nm.

2.10. Cell culture study

Cell culture was carried out using commercial mouse fibroblasts L929 (NCTC clone 929, ATCC® CCL-1™). Cells were cultured on tissue culture flasks as per manufacturers' instructions, then trypsinised with Tryple Express to detach from the culture surfaces. The cells were centrifuged for 3 min at 0.3 g and the supernatant discarded. For cell culture studies, fibroblasts were resuspended in **PEG-(SQ)$_8$** solution (c = 5 mM) to achieve a cell density of 5 × 10^6 cells per mL. The solution was agitated gently to allow the cells to distribute throughout the solution and pipetted into tissue culture inserts. The inserts were exposed to green light (λ = 505 nm, I = 20 mW cm^{-2}) irradiation for 30 minutes and cell culture media (Dulbecco's Modified Eagle Medium) were added. Triplicates were prepared. The cell-laden hydrogel samples were rinsed twice with culture media and maintained at 37 °C and 5% CO_2. At day 1 cell culture media were exchanged once. To assess cell viability, gels were removed from tissue culture inserts after day 1 and day 3 in culture, washed in PBS and stained using Live/Dead® Viability/Cytotoxicity Kit for mammalian cells (Invitrogen) following the manufacturer's recommended protocol.

2.11. Synthetic procedures

2.11.1 Materials

Solvents (CH_2Cl_2, diethyl ether, petroleum ether, acetone, tetrahydrofuran, and methanol) were purchased from VWR in HPLC grade and used directly. 8arm PEG-OH (MW = 20 000 Da) was purchased from Jenkem Tech, USA. MeO-PEG-NH_2 was prepared according to a previously published procedure with the degree of conversion from -OH to -NH2 group of 97%.[6] All other chemicals were purchased from Sigma-Aldrich and used as received. All synthesized compounds were stored in a freezer at -20 °C and covered with aluminium foil.

2.11.2 Synthesis of ethyl 4-(4-formyl-2-methoxyphenoxy) butanoate (S1)

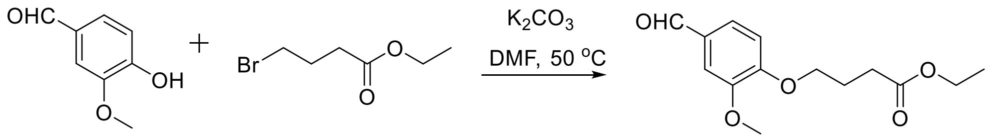

S1

Ethyl 4-bromobutyrate (6.44 g, 0.033 mol) and vanillin (4.56 g, 0.03 mol) were dissolved in DMF (50 mL). K_2CO_3 (6.9 g, 0.05 mol) was added and the mixture was heated at 50 °C under stirring for 2 h. The mixture was then filtered, diluted with water (50 mL) and extracted with ethyl acetate (100 mL). The organic extract was washed with water (100 mL x 2), brine (100 mL), dried ($MgSO_4$) and concentrated in vacuo to give product as white solid (yield: 6.9 g, 87%). ^1H NMR data are in agreement with published procedure.[7]

2.11.3 Synthesis of 2-methylquinoxaline (S2)

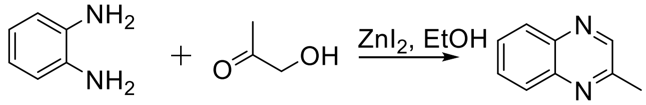

S2

Methylglyoxal (40% in water, 5.4 g, 0.03 mmol) was added to a solution of benzene-1,2-diamine (2.16 g, 0.02 mol) and ZnI_2 (1.27 g, 4 mmol) in ethanol/water (20 mL/20 mL) and the solution was heated at 80 °C for 1 h. The solution was then partially concentrated in vacuo, extracted with CH_2Cl_2 (50 mL x 2), dried ($MgSO_4$) and concentrated in vacuo. The crude product was purified by column chromatography eluting with hexane: ethyl (v/v = 7/3) to give product as yellow liquid (yield: 2.56 g, 89%). ^1H NMR (δ / ppm): 8.76, s; 8.02-8.1, m; 7.72-7.76, m; 2.8, s.

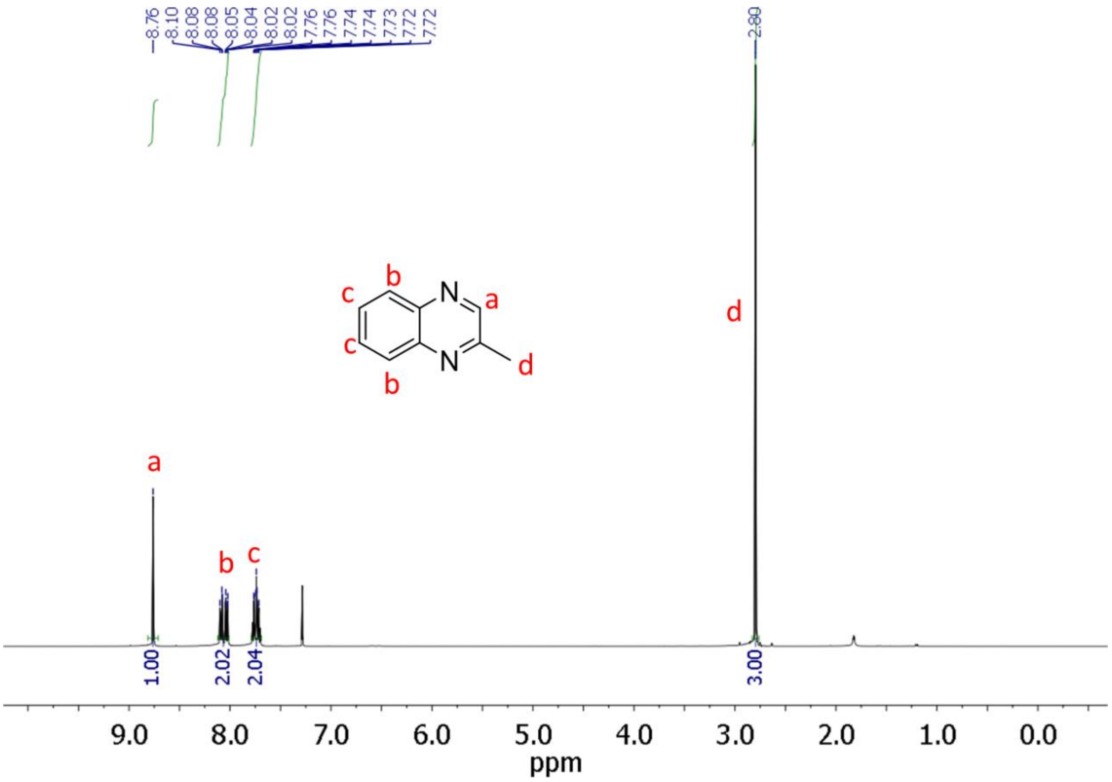

Supplementary Figure 15. ^{1}H NMR spectrum of compound **S2** ($CDCl_3$, 400 MHz).

2.11.4 Synthesis of ethyl (E)-4-(2-methoxy-4-(2-(quinoxalin-2-yl)vinyl)phenoxy)butanoate (S3)

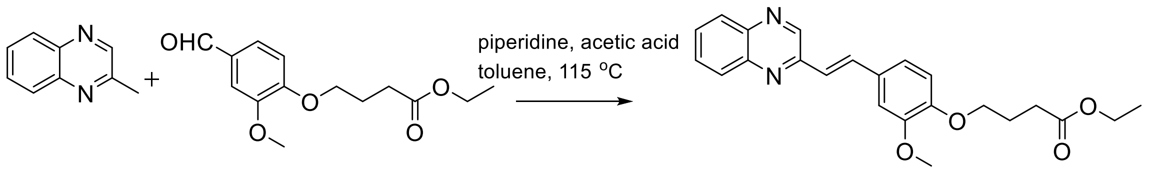

S3

To a mixture of **S1** (1.44, 0.01 mol) and **S2** (4 g, 0.015 mol) was added piperidine (0.425 g, 5 mmol), acetic acid (0.42 g, 7 mmol) and dry toluene (5 mL). The mixture was purged with Argon, sealed and heated at 115 °C for 48 h. The resultant solution was concentrated in vacuo and absorbed onto silica gel. The product was purified by column chromatography running with hexane: ethyl acetate (v/v = 4/6) to give pure product as yellow solid (yield: 2.78 g, 71%). ^{1}H NMR (δ / ppm): 9.07, s; 8.06-8.1, t, *J* = 6.95 Hz; 7.81-7.85, d, *J* = 16.4 Hz; 7.71-7.78, m; 7.3-7.3, m; 6.93-6.95, d, *J* = 8.19 Hz; 4.13-4.2, m; 3.87, s; 2.56-2.59, t, *J* = 7.28 Hz; 2.19-2.23, m; 1.27-1.3, t, *J* = 7.1 Hz.

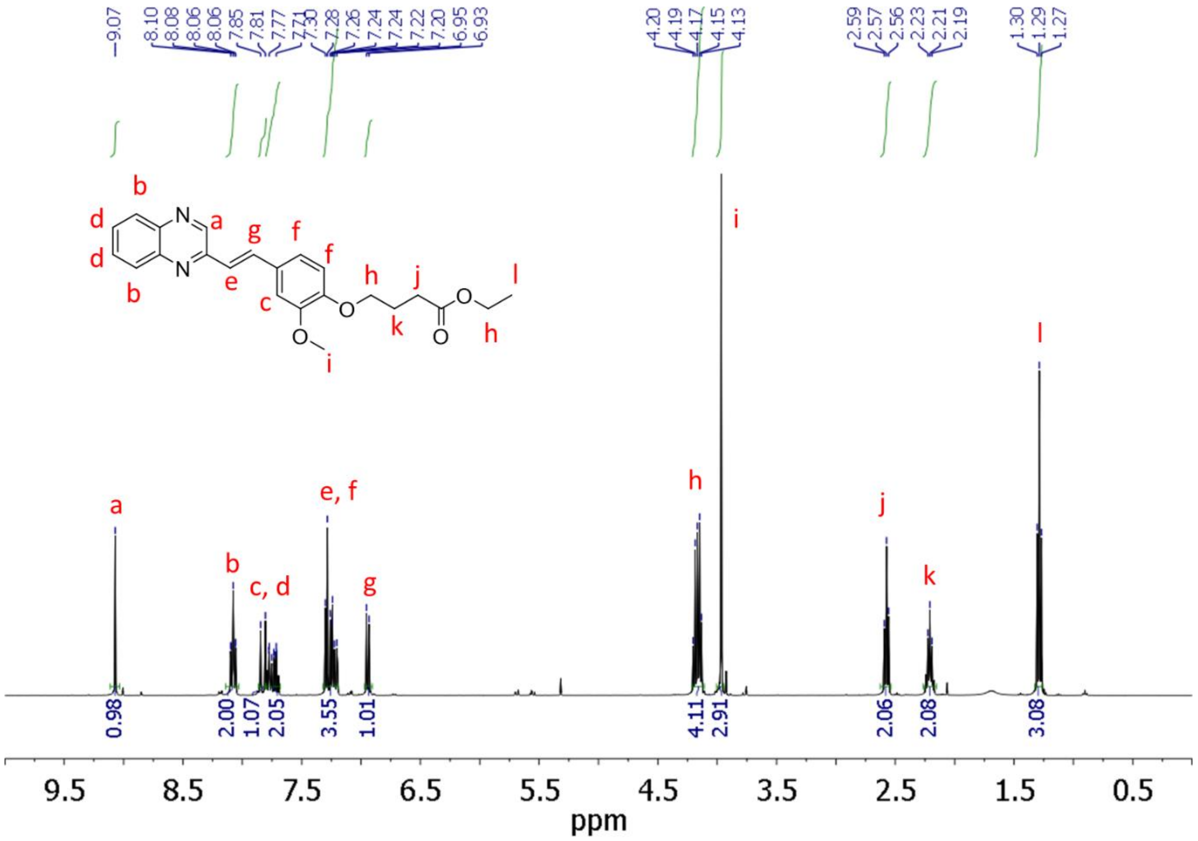

Supplementary Figure 16. ^{1}H NMR spectrum of compound **S3** ($CDCl_3$, 400 MHz).

2.11.5 Synthesis of (E)-4-(2-methoxy-4-(2-(quinoxalin-2-yl)vinyl)phenoxy)butanoic acid (S4)

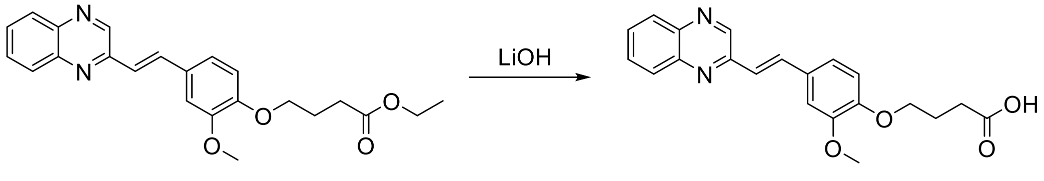

S4

Compound **S3** (1.96 g, 5 mmol) was dissolved in THF (20 mL), to this solution was added a solution of LiOH (0.239 g, 10 mmol) in water (20 mL) and the solution was stirred at ambient temperature for 1 h. The solution was partially concentrated in vacuo and pH was adjusted to ca. 6.5 using HCl 1M solution. The solution was further diluted with water (100 mL), filtered and dried in vacuo to give product as yellow solid (yield: 1.34 g, 73.6%). ^{1}H NMR (δ / ppm): 8.98, s; 7.97-7.99, t, J = 6.95 Hz; 7.63-7.75, d, J = 16.4 Hz; 7.65-7.73, m; 7.3-7.3, m; 6.93-6.95, d, J = 8.19 Hz; 4.08-4.12, m; 3.81, s; 2.83-2.85, t, J = 7.28 Hz; 2.21-2.25, m.

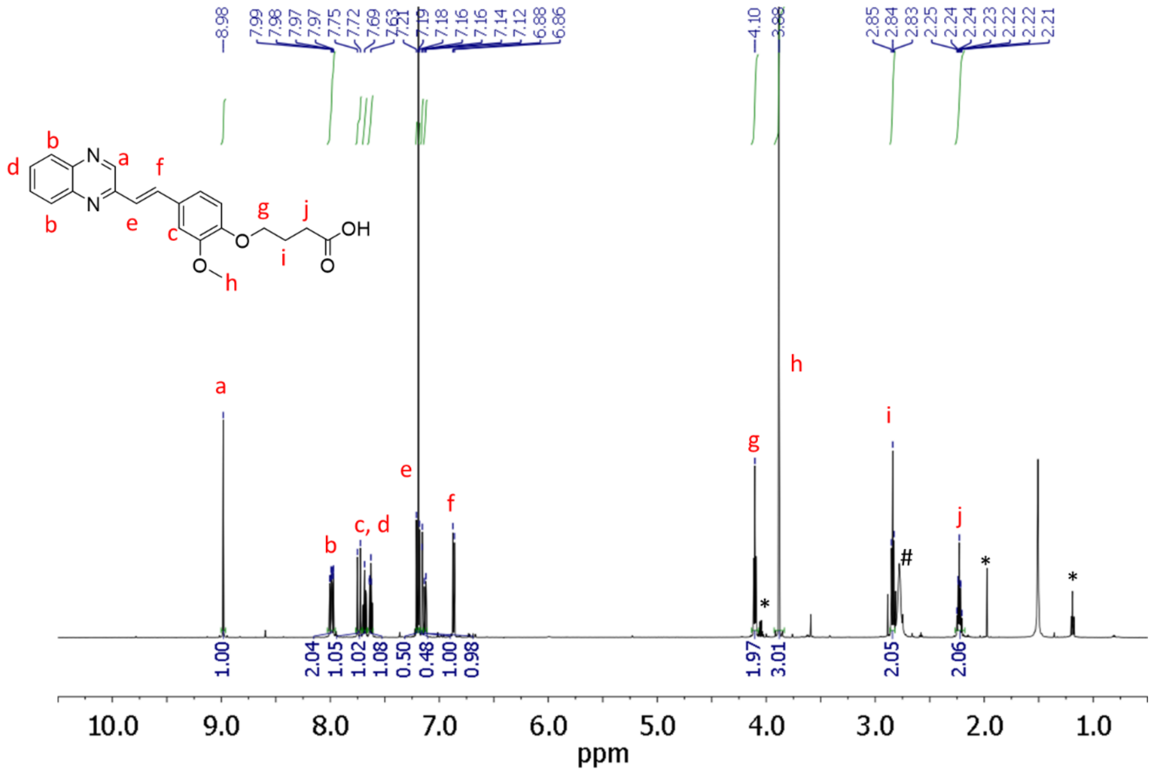

Supplementary Figure 17. ^{1}H NMR spectrum of compound **S4** ($CDCl_3$, 600 MHz; *: ethyl acetate, #: water).

2.11.6 Synthesis of 2,5-dioxopyrrolidin-1-yl (E)-4-(2-methoxy-4-(2-(quinoxalin-2-yl)vinyl)phenoxy)butanoate (S5)

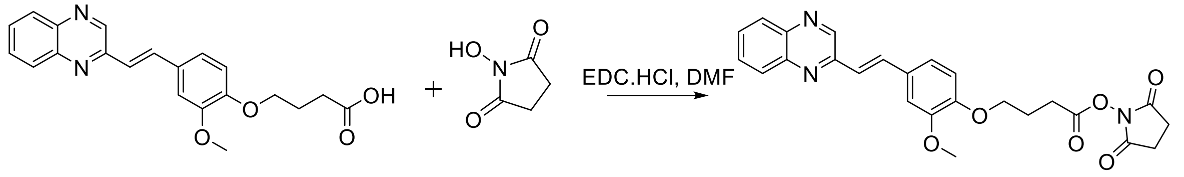

Compound **S4** (0.728 g, 2 mmol) was dissolved in DMF (10 mL) and to this solution was added N-(3-Dimethylaminopropyl)-N′-ethylcarbodiimide hydrochloride (EDC.HCl, 0.478 g, 2.5 mmol) and N-Hydroxysuccinimide (0.288 g, 2.5 mmol) and the solution was stirred at ambient temperature for 6 h. DMF was concentrated in vacuo and the residue was absorbed onto silica gel (*ca.* 1.5 g). The product was purified by column chromatography running with cyclohexane: ethyl acetate (v/v = 3/7) to give product as yellow solid (yield: 0.32 g. 34.7%). ^{1}H NMR (δ / ppm): 9.01, s; 8-8.04, t, J = 6.95 Hz; 7.68-7.79, d, J = 16.4 Hz; 7.59-7.68, m; 7.3-7.3, m; 6.86-6.91, d, J = 8.19 Hz; 4.09-4.12, m; 3.88, s; 2.82-2.86, t, J = 7.28 Hz; 3.94, s; 2.2-2.25, m.

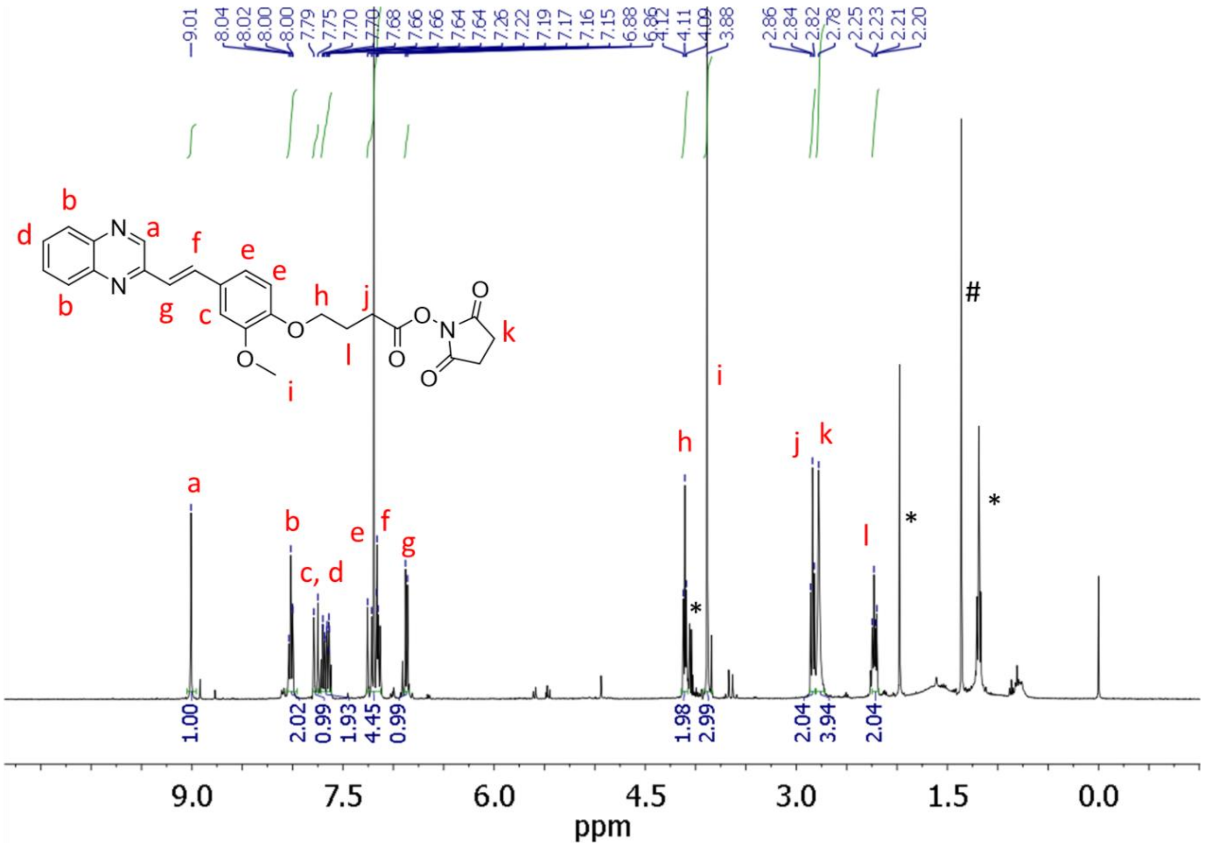

Supplementary Figure 18. ^{1}H NMR spectrum of compound S5 ($CDCl_3$, 400 MHz; *: ethyl acetate, #: cyclohexane).

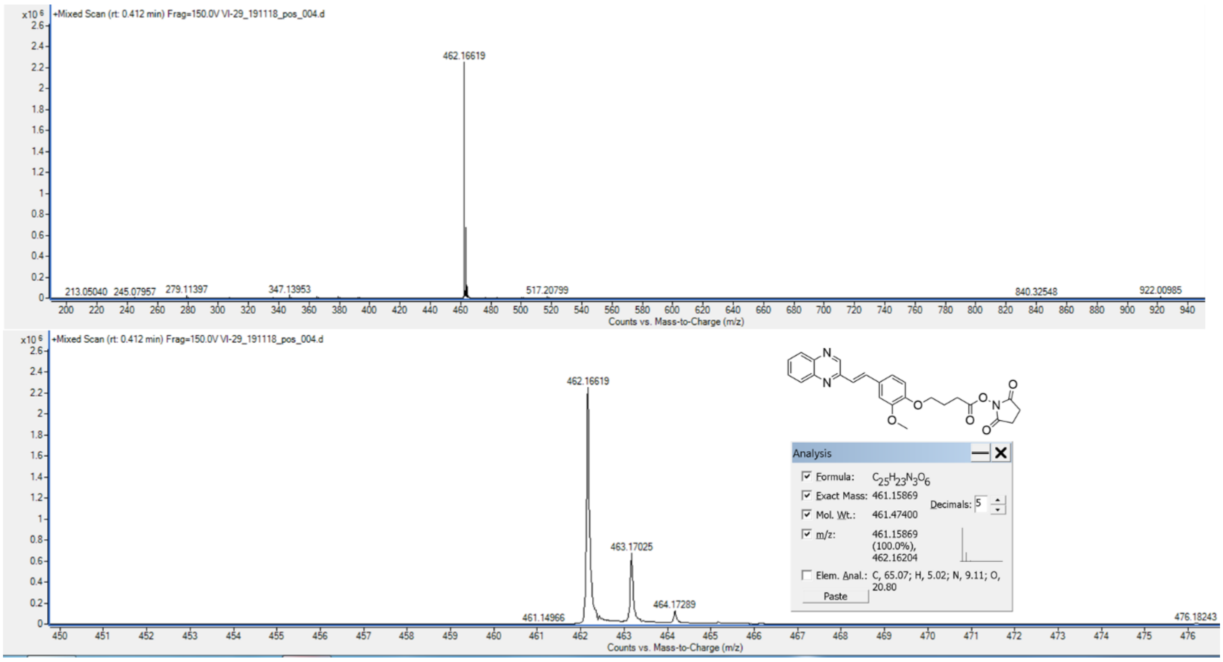

Supplementary Figure 19. HRMS spectra of compound **S5**.

2.11.7 Synthesis of 8-arm PEG-NH_2

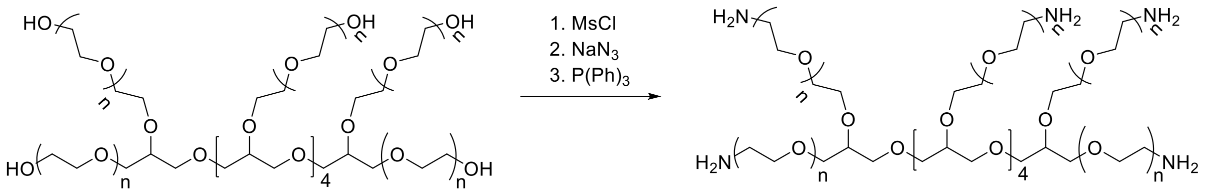

8-arm PEG20k (5 g, 0.25 mmol) was dissolved in CH_2Cl_2 (20 mL). Triethylamine (1.2 g, 12 mmol) was added and the solution was cooled on an ice bath. Methansulfonyl chloride (1.14 g, 10 mmol) was added dropwise over 30 min and the solution was allowed to warm to room temperature. The solution was stirred for 2 h, filtered and precipitated into diethyl ether (200 mL) to give white powder that was used directly in the next step.

The above product was dissolved in DMF (10 mL) and sodium azide (1.3 g, 20 mmol) was added and the solution was stirred at 80 °C for 10 h. DMF was concentrated in vacuo and the residue was taken up in dichloromethane (50 mL), and filtered. The filtrate was washed with water (100 mL x2), brine (100 mL), dried (MgSO4), concentrated in vacuo to ca. 10 mL and precipitated into diethyl ether (200 mL) to give white polymer that was used directly in the next step.

The above product was dissolved in methanol (50 mL) and triphenylphosphine (2.62 g, 10 mmol) was added. The resultant solution was heated at 80 °C under refluxing condition overnight. The solution was then concentrated in vacuo and water (50 mL) was added. The mixture was then extracted with diethyl ether (50 mL x 3) and the organic phase was discarded. The aqueous phase was then extracted with dichloromethane (50 mL x2). The combined organic phases were dried (MgSO4), concentrated in vacuo to ca. 10 mL and precipitated into diethyl ether (200 mL) to give product as white powder (total yield: 3.8 g, *ca.* 76%). ^{1}H NMR (δ / ppm): 3.81-3.83, m; 3.65, broad s, 3.56-3.48, t, J = 4.6 Hz; 2.91-2.93, t, J= 5.25 Hz.

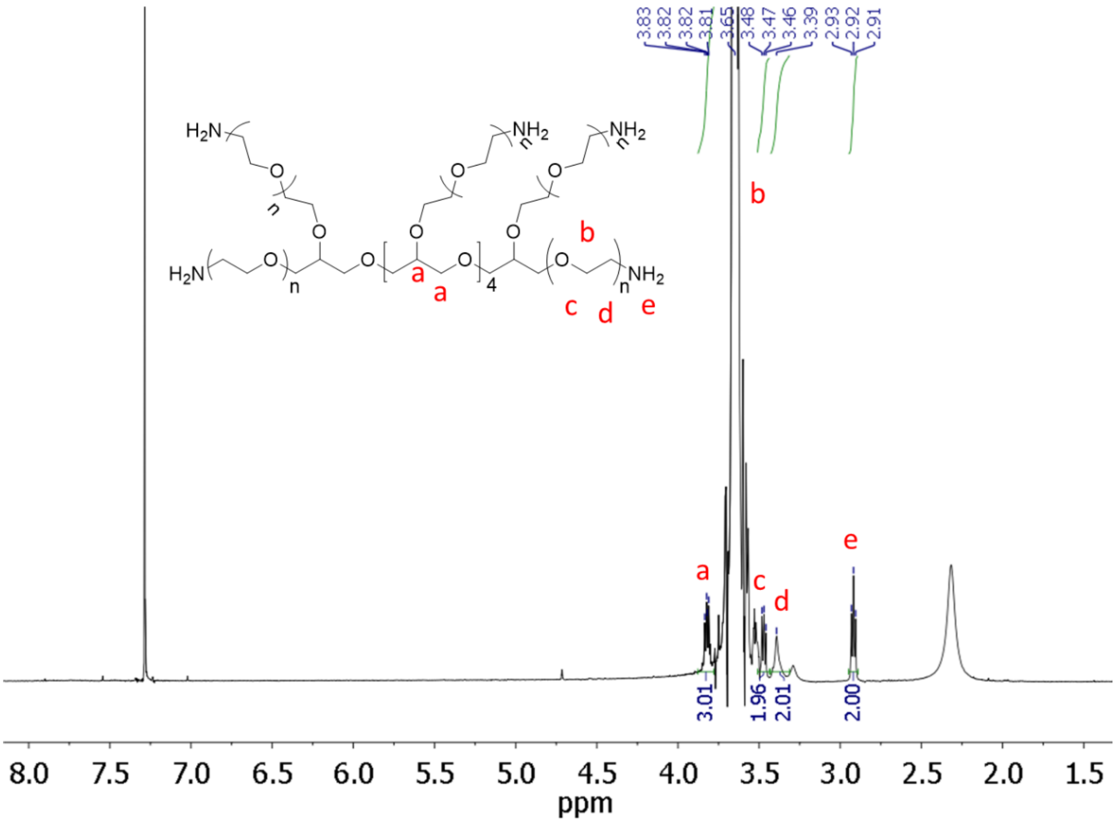

Supplementary Figure 20. ^{1}H NMR spectrum of 8-arm PEG-NH_2 ($CDCl_3$, 400 MHz).

2.11.8 Synthesis of linear MeO-PEG-styrylquinoxaline (PEG-SQ) and 8arm-PEG-styrylquinoxaline (8arm $(PEG\text{-}SQ)_8$)

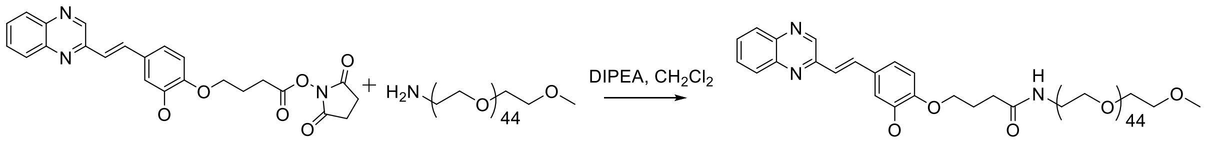

MeO-PEG-NH_2 (1 g, 0.5 mmol) was dissolved in CH_2Cl_2 (5 mL), to this solution was added **S5** (230.5 mg, 0.5 mmol) and N,N-Diisopropylethylamine (DIPEA, 20 µL). The solution was stirred at ambient temperature for 2 h and precipitated into diethyl ether (100 mL) to give product as yellow powder (yield: 0.97 g, ca. 80%).

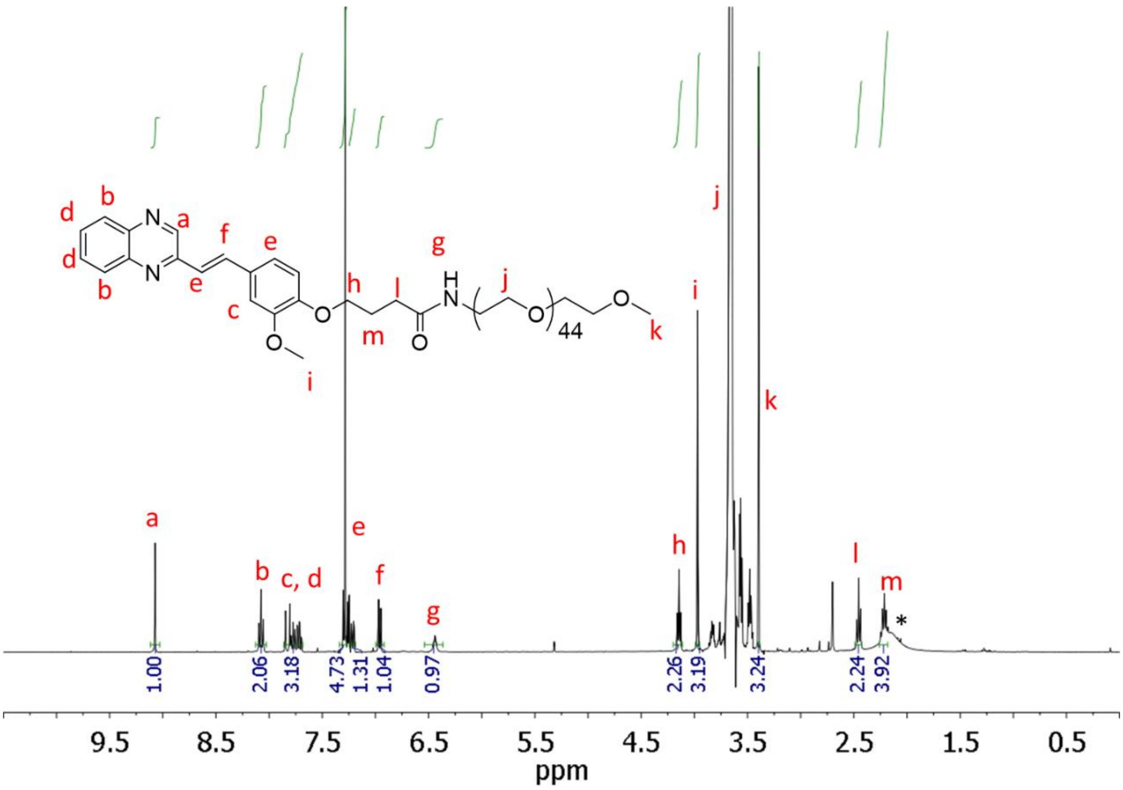

Supplementary Figure 21. ^{1}H NMR spectrum of **P1** ($CDCl_3$, 400 MHz).

8arm-PEG- styrylquinoxaline (8arm **(PEG-SQ)₈**) was synthesized using similar procedure.

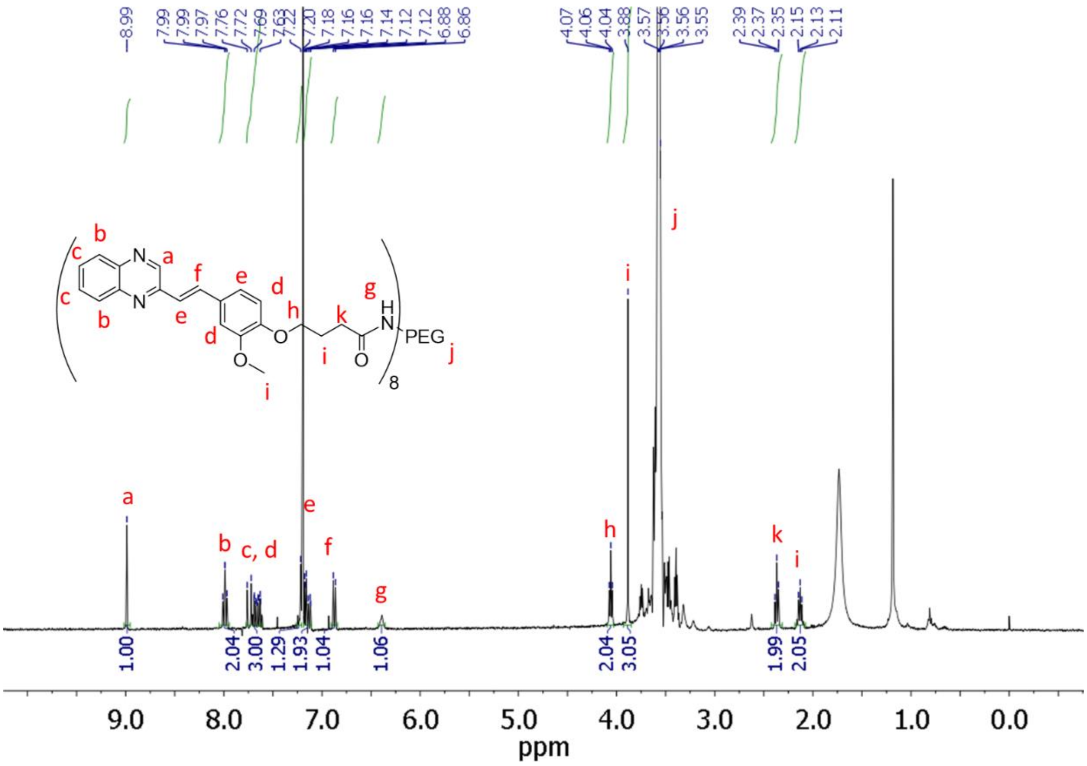

Supplementary Figure 22. ^{1}H NMR spectrum of **P2** ($CDCl_3$, 400 MHz).

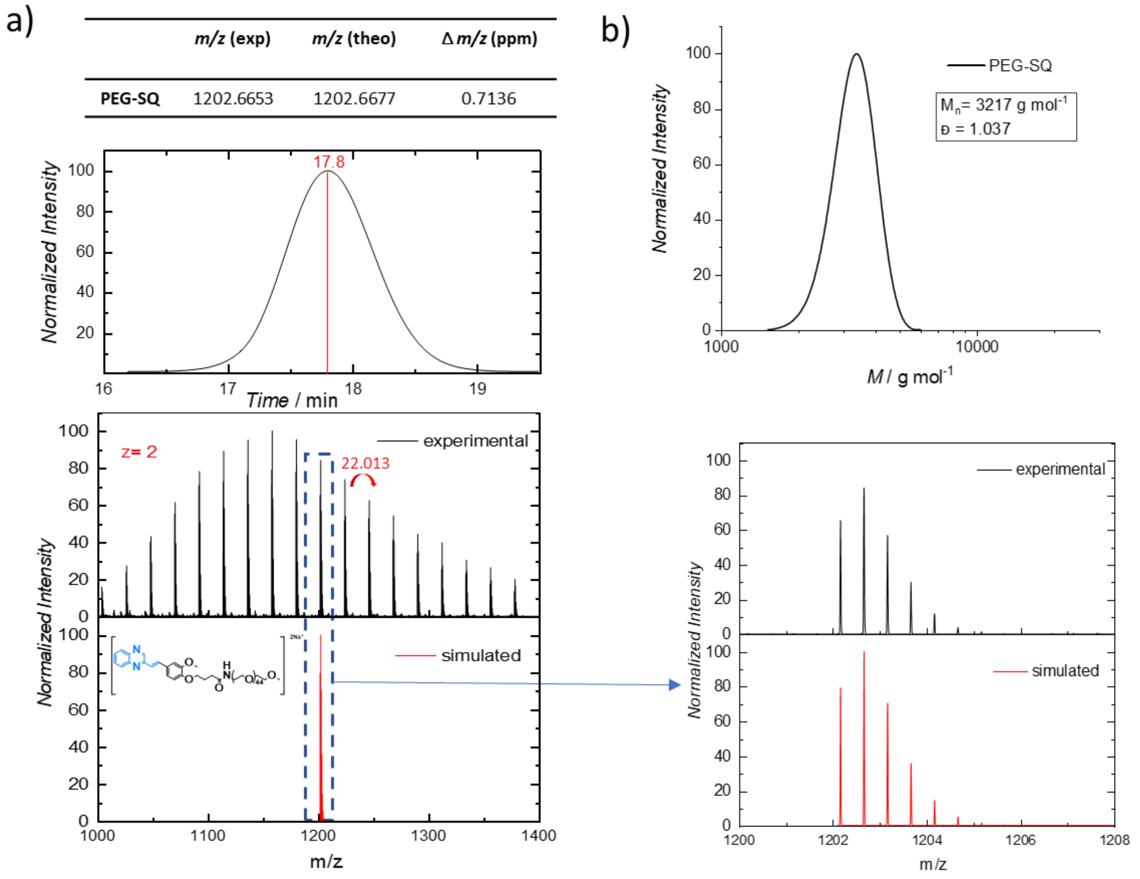

	m/z (exp)	*m/z* (theo)	Δ *m/z* (ppm)
**PEG-SQ**	1202.6653	1202.6677	0.7136

Supplementary Figure 23. a) SEC-MS analysis of **PEG-SQ**. Table shows theoretical and calculated *m/z* values. Top graph shows SEC (THF) trace. Bottom two graphs show MS trace of experimental and simulated doubly charged **PEG-SQ** patterns. *m/z* difference between experimental patterns equals to 22.013 which corresponds to *m/z* value of one PEG repeating unit. **b)** SEC (DMAC) data (calibrated against PMMA) of **PEG-SQ**.

3. Supplementary References

1. Gruendling, T.; Guilhaus, M.; Barner-Kowollik, C. *Macromolecules* **42,** 6366-6374 (2009).
2. Marschner, D. E.; Frisch, H.; Offenloch, J. T.; Tuten, B. T.; Becer, C. R.; Walther, A.; Goldmann, A. S.; Tzvetkova, P.; Barner-Kowollik, C. *Macromolecules* **51,** 3802–3807 (2018).
3. Menzel, J. P.; Noble, B. B.; Lauer, A.; Coote, M. L.; Blinco, J. P.; Barner-Kowollik, C. *J. Am. Chem. Soc.* **139,** 15812-15820 (2017).
4. Fast, D. E.; Lauer, A.; Menzel, J. P.; Kelterer, A.-M.; Gescheidt, G.; Barner-Kowollik, C. *Macromolecules* **50,** 1815-1823 (2017).
5. Menzel, J. P.; Feist, F.; Tuten, B.; Weil, T.; Blinco, J. P.; Barner-Kowollik, C. *Angew. Chem. Int. Ed.* **58,** 7470-7474 (2019).
6. Kalayci, K.; Frisch, H.; Barner-Kowollik, C.; Truong, V. X. *Adv. Funct. Mater.* 1908171 (2020).
7. Wada, K.; Lee, J. Y.; Hung, H. Y.; Shi, Q.; Lin, L.; Zhao, Y.; Goto, M.; Yang, P. C.; Kuo, S. C.; Chen, H. W.; Lee, K. H. *Bioorg. Med. Chem.* **23,** 1507-1514 (2015).
