# Supplementary material for: Green light triggered [2+2] cycloaddition of halochromic styrylquinoxaline—controlling photoreactivity by pH
Source: Nat Commun. 2020 Aug 21;11:4193. doi: 10.1038/s41467-020-18057-9 (PMC7443129; doi:10.1038/s41467-020-18057-9)
Supplement: Supplementary file 3 — Description of Additional Supplementary Files [file 41467_2020_18057_MOESM3_ESM.pdf]

Description of Additional Supplementary Files

File Name: Supplementary Movie 1
Description: halochromic effect of 8-arm PEG-SQ solution, color change from acidic to neutral pH

File Name: Supplementary Movie 2
Description: Effect of pH on photo-crosslinking – gelation happened under green light when pH was switched from acidic to neutral
